# The prevalence of atopic dermatitis beyond childhood: A systematic review and meta‐analysis of longitudinal studies

**DOI:** 10.1111/all.13320

**Published:** 2017-11-24

**Authors:** K. Abuabara, A. M. Yu, J.‐P. Okhovat, I. E. Allen, S. M. Langan

**Affiliations:** ^1^ Program for Clinical Research Department of Dermatology University of California San Francisco (UCSF) San Francisco CA USA; ^2^ Faculty of Medicine University of Ottawa Ottawa ON Canada; ^3^ Harvard T.H. Chan School of Public Health, Beth Israel Deaconess Medical Center, and Harvard Medical School Boston MA USA; ^4^ Department of Epidemiology and Biostatistics University of California San Francisco (UCSF) San Francisco CA USA; ^5^ Faculty of Epidemiology & Population Health London School of Hygiene and Tropical Medicine London UK

**Keywords:** atopic dermatitis, atopic eczema, eczema, natural history, prevalence

## Abstract

**Background:**

There are sparse and conflicting data regarding the long‐term clinical course of atopic dermatitis (AD). Although often described as a childhood disease, newer population‐based estimates suggest the prevalence of pediatric and adult disease may be similar.

**Methods:**

Our objective was to determine whether there is a decline in the prevalence of AD in population‐based cohorts of patients followed longitudinally beyond childhood. We conducted a systematic review and meta‐analysis including studies assessing AD prevalence across 3 or more points in time. The primary outcome was weighted overall risk difference (percentage decrease in AD prevalence).

**Results:**

Of 2080 references reviewed, 7 studies with 13 515 participants were included. Participants were assessed at 3‐6 time points, ranging from age 3 months to 26 years. The percentage decrease in prevalence after age 12 was 1%, which was not significantly different from zero (95% confidence interval −2%‐5%). Similar results were found with other age cut‐offs.

**Conclusion:**

The prevalence of AD in longitudinal birth cohort studies is similar in childhood and adolescence/early adulthood.

## 1 INTRODUCTION

Atopic dermatitis (AD), also known as atopic eczema or simply eczema,^1^ is one of the most common and burdensome diseases of childhood, yet little is known about the long‐term clinical course of the disease.^2^ Textbooks and review articles suggest that most individuals develop disease within the first 2 years of life, experience episodic symptoms throughout childhood, and improve by adolescence; yet, sparse and conflicting data exist regarding the proportion of individuals whose disease resolves and little is known about predictors of disease persistence or adult‐onset disease.^3^, ^4^, ^5^ Challenging the traditional notion of AD as a predominantly childhood disease, a growing body of research on the pathophysiology of AD points to genetic causes of altered skin barrier and immune dysfunction that could predispose to episodic disease throughout life.^6^, ^7^ Additionally, population‐based estimates from cross‐sectional surveys suggest that AD may be as common in adults as in children.^8^ If AD prevalence does not decline over time, there are important implications for patient counseling, management, and future research.

The clinical course of AD has been particularly challenging to study because the condition is heterogeneous and intermittent. Individuals have different clinical presentations, and many will have periods without symptoms or skin lesions. Clinical trials generally focus on short‐term disease control, and cross‐sectional studies offer a snapshot of the population and hence cannot be used to generate prognostic information for individuals. Thanks to growing interest in atopic diseases, a number of longitudinal cohort studies included measures of AD disease activity at multiple time points in the same individuals, enabling the estimation of changes in AD prevalence as a given population ages. The primary objective of this study was to systematically review and analyze longitudinal studies of AD that include repeated measurements in the same cohort during and after childhood to test the hypothesis that the prevalence of AD does not decrease with age.

## 2 METHODS

### 2.1 Data source and searches

This review is reported in accordance with the Preferred Reporting Items for Systematic Reviews and Meta‐Analyses (PRISMA) and Meta‐analysis of Observational studies in Epidemiology (MOOSE) guidance.^9^, ^10^ Our research protocol was registered on PROSPERO and was publically available prior to the study start date (registration ID: 42016033553). PubMed and EMBASE were searched from database inception to October 2015 using prespecified search terms, MeSH (medical subject heading) headings and keywords, developed in consultation with a professional medical librarian. Search terms are described in Table S1. Reference lists of included studies were also screened for additional potentially relevant articles.

This study was exempt from IRB review because it included only published data and is not considered Human Subjects Research by the UCSF IRB (http://irb.ucsf.edu/not-human-subjects-research).

### 2.2 Study selection

We included studies of patients with AD as diagnosed by a physician or using a standardized definition such as the UK working party diagnostic criteria for AD or the International Study of Asthma and Allergies in Children ISAAC criteria.^11^, ^12^ Because our objective was to examine changes in AD prevalence within a given cohort as populations aged, we included only longitudinal cohort studies that assessed AD activity in the same patients at 3 or more distinct time points, to account for the episodic nature of AD and allow for observation of any nonlinear patterns of change. Further, to capture AD prevalence beyond childhood, studies were required to include at least 1 assessment with participants over the age of 12 years. We chose this cutoff because prior studies suggested high rates of “remission” in adolescence and a longitudinal prospective study suggested that prevalence declines most rapidly between ages 8 and 11.^13^, ^14^ We excluded studies that were clinic‐based, or focused on patients with localized forms of AD (eg, hand eczema). We did not select studies on the basis of interventions and our search included studies in all available languages.

### 2.3 Data extraction and quality assessment

Title and abstract screening followed by full‐text screening was performed independently and in duplicate to ascertain whether studies met eligibility criteria. Extracted variables included study information (authors, year of publication); cohort information (country of study, cohort name, method of participant recruitment); study size (number of patients at study start/end, and alternate measures of loss to follow‐up); patient characteristics (sex, race/ethnicity, other demographic information); AD diagnostic criteria; and finally, method and age of AD prevalence assessment.

Methodological quality was assessed using the Newcastle‐Ottawa Scale for cohort studies.^15^ The risk of bias and scoring criteria used in the present review are described in Table S2. Screening, data extraction, and risk of bias assessment were all performed in duplicate by 2 authors (AY and JO), and discrepancies were resolved by group consensus (AY, JO, KA, and SML).

### 2.4 Data synthesis and analysis

The primary outcome was percentage change in AD prevalence by age, and all measured time points were included. Secondary outcomes included the prevalence of other atopic conditions (asthma, rhinitis/hayfever), treatment information, and severity data. Corresponding authors of included studies were contacted via e‐mail to verify extracted study prevalence data used for the analyses.

We tabulated and plotted the AD prevalence by age for each study. We then calculated the standardized difference in mean prevalence before and after the age of 12 years (using all time points from each study), and report individual study differences and the mean weighted risk difference. We tested for heterogeneity with the chi‐square test and measured inconsistency using the I^2^ statistic, which represents the percentage of total variation across studies.^16^ Because of the methodologic and clinical heterogeneity inherent in the data, we used random‐effects models for all meta‐analyses.

Preplanned sensitivity analyses were conducted to assess the impact of varying the age cutoffs. The primary analysis was repeated using different age cutoffs: <10 years vs ≥10 years; <8 years vs ≥8 years. These analyses were performed to test whether our choice of an age 12 cutoff affected the results. We also repeated the analysis comparing those aged <2 years to 3‐11 years and those aged 2‐12 years vs >12 years to test whether including very young children (when the diagnosis of AD can be more challenging) affected our results. Additional stratified analyses were performed to explore sources of heterogeneity, including analyses by age, by country, by loss to follow‐up, by the number of measurements, and a “jackknife” analysis that eliminated 1 study at a time. Egger's test was used to evaluate for publication bias.^17^ All analyses were performed with Stata (version 14, Stata Corporation, College Station, TX, USA).

## 3 RESULTS

**Figure 1:**
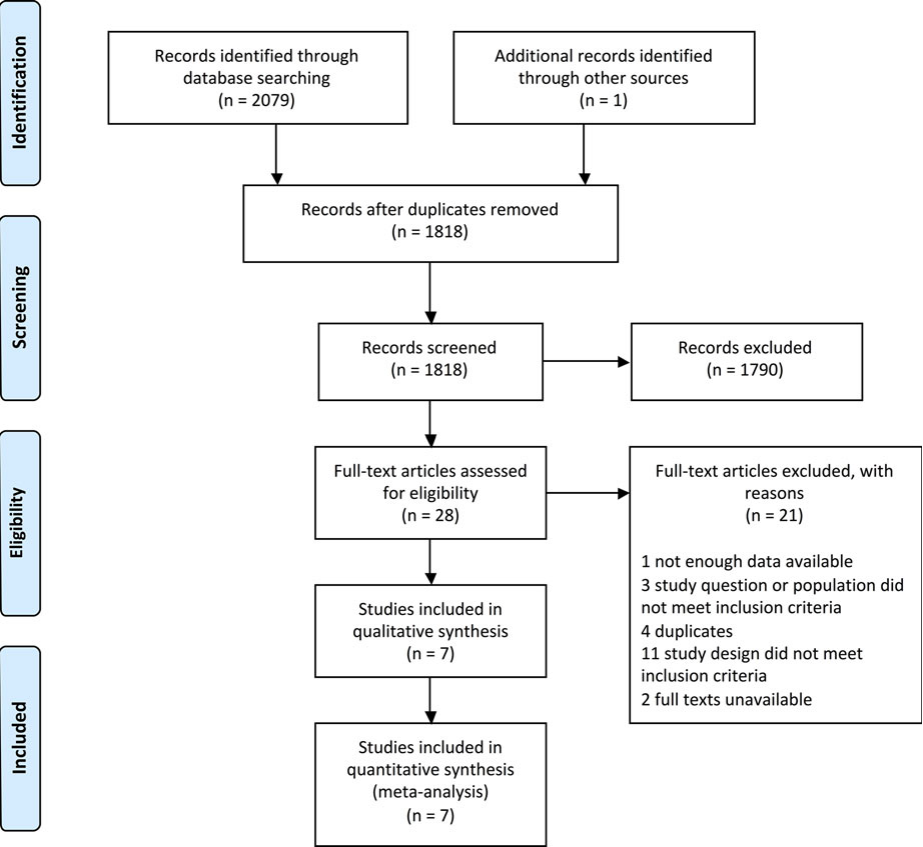
PRISMA flow diagram. Results of search strategy [Colour figure can be viewed at wileyonlinelibrary.com]

The search yielded 2080 records. After full‐text review of 28 manuscripts, 7 studies were selected for inclusion.^14^, ^18^, ^19^, ^20^, ^21^, ^22^, ^23^ The study flow diagram (Figure 1) lists the reasons why 21 studies were excluded.

### 3.1 Study characteristics

Table 1 outlines the characteristics of each study in detail. The included studies followed 13 515 individuals. All geographical locations could be broadly classified as Northern European: Studies were conducted in Sweden, Iceland, Germany, Denmark, and the UK (3 studies). All studies began follow‐up at birth, and most were initiated the 1980s, with the exception of Williams et al's study which began follow‐up in 1958, and Ballardini et al, which began follow‐up in 1994‐1996. Each study measured prevalence at 3‐6 separate time points, which ranged between patient ages of 3 months and 26 years. The annual period prevalence of AD was assessed via questionnaire in all studies, with the addition of physical examination at some time points in 3 studies. The diagnostic criteria varied slightly by study and are outlined in detail in Table S3. Data were largely unavailable for the secondary outcomes: Only 4 studies reported data on the prevalence of other atopic outcomes, and the methods for assessment and reporting differed. None of the studies reported data on treatment or severity of AD (Table 1).

**Table 1:** Summary of study characteristics of included studies

Study	Country	Longitudinal cohort		Number of patients (N)			Sex (% male)	Prevalence			Other atopic outcomes reported (Y/N)	Treatment reported (Y/N)	Severity reported (Y/N)
		Study cohort name	Year of birth	Study start	Study end	Loss to follow‐up		Time points (years)	Method of assessment	Period prevalence			
Ballardini et al^18^	Sweden	BAMSE birth cohort	1994‐1996	2916	2916	29%^b^	N/R	1, 2, 4, 8, 12	Questionnaire	1 y	Y	N	N
Burr et al^19^	UK	Not reported	1982‐1984	497	304	39%	N/R	0.25, 0.5, 1, 7, 15, 23	Clinical examination (until the age of 7), Questionnaire	1 y	N	N	N
Finnbogadóttir et al.^20^	Iceland	Not reported	1987	179	120	33%	N/R	2, 4, 8, 16, 21	Clinical examination, Questionnaire	1 y	Y	N	N
Gough et al^21^	Germany	MAS‐90 birth cohort	1990	1314	942	28%	52%	3, 6, 9, 12, 15, 20	Questionnaire	1 y	Y	N	N
Nissen et al^22^	Denmark	Not reported	1985	276	193	30%	47%	2, 5, 10, 15, 26	Clinical examination	1 y	Y	N	N
Williams et al^14^	UK	National Child Development Study	1958	6877	6877	53%^b^	N/R	1‐7, 11, 16, 23	Clinical examination, Questionnaire	1 y^b^	N	N	N
Ziyab et al^23^	UK	1989 Isle of Wight birth cohort	1989	1456	1307	10%	N/R	1, 2, 4, 10, 18	Questionnaire	1 y	N	N	N

UK: United Kingdom; Y: yes; N: no; y: year.

a The publication only included those with complete data on all health outcomes at every follow‐up, which represented 71% of the original cohort.

The publication only included those with complete data on all health outcomes at every follow‐up, which represented 47% of the original cohort.

b At the age of 7, participants were asked about any AD up to the age of 7, at subsequent ages they were asked about AD over the past year.

### 3.2 Prevalence estimates

**Figure 2:**
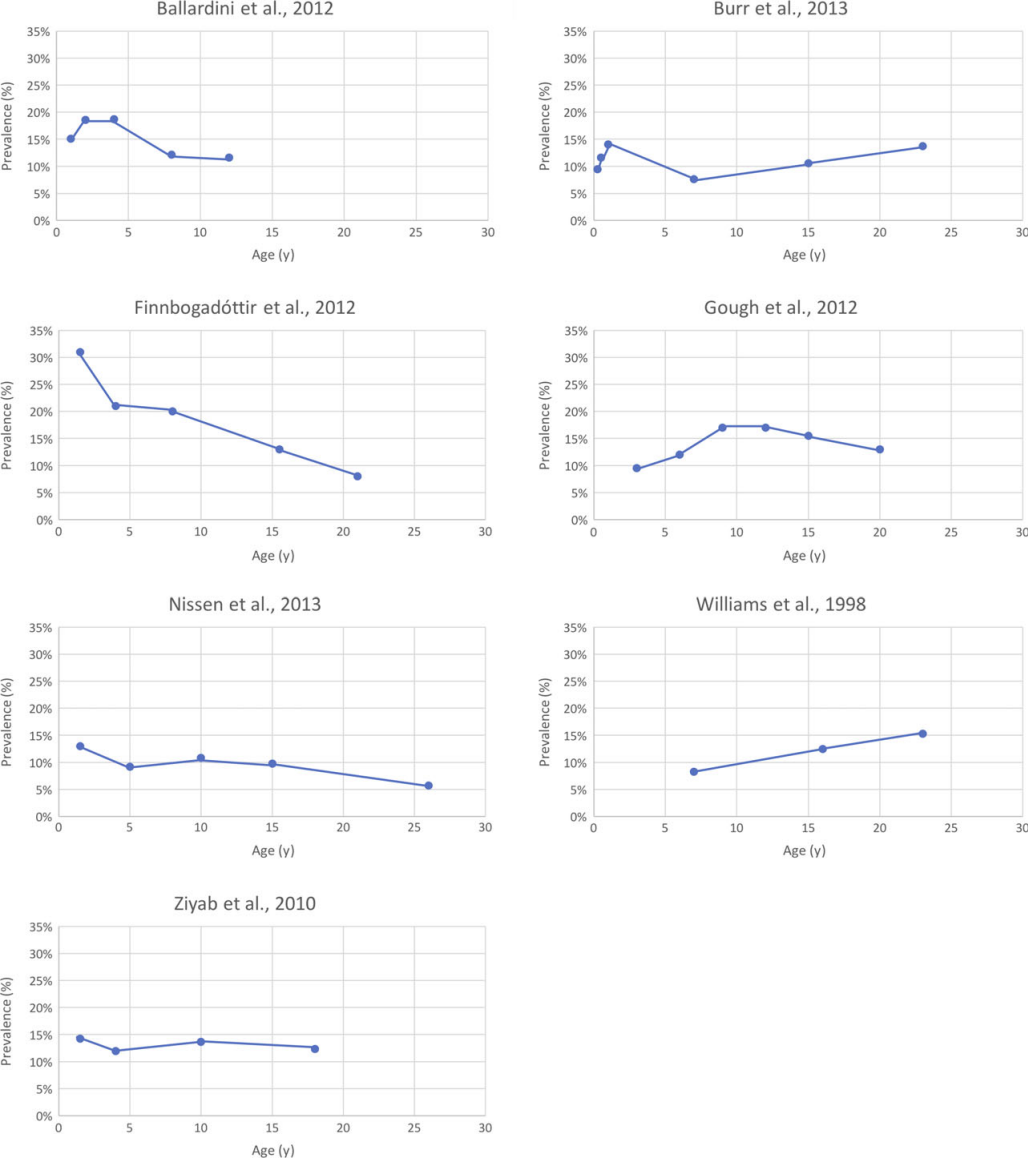
Longitudinal prevalence estimates of included studies. Proportion of the population with AD at each age [Colour figure can be viewed at wileyonlinelibrary.com]

The annual period prevalence of AD ranged from 6% at the age of 26 years^22^ to 34% at the age of 12 years.^21^ Study‐specific figures are presented graphically in Figure 2, and the exact numbers used in the meta‐analysis are included in Table S4.

Data on gender were inconsistent. Gough et al observed that AD prevalence tended to increase with age in the female population, and decrease among males. Similarly, in Burr et al and Ziyab et al, AD prevalence in females rose from age of 15 to 23 years and 10 to 18 years, respectively, while declining in males over the same time period. In contrast, Finnobogadottir et al, and Ballardini et al, did not find any difference between AD prevalence between males or females across all time points.

### 3.3 Risk difference

**Figure 3:**
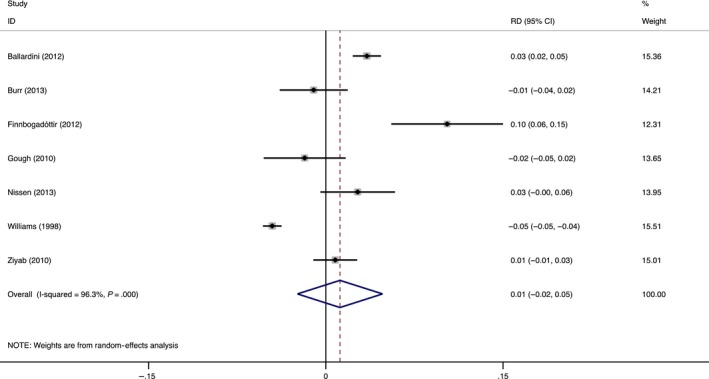
Results of meta‐analysis of risk difference in AD prevalence. Risk difference (RD) in prevalence in children age <12 years of age compared with children age ≥12 years [Colour figure can be viewed at wileyonlinelibrary.com]

The primary outcome was the percentage change in prevalence after the age of 12 years. This ranged from −0.05 (meaning there was a 5% increase in the prevalence after the age of 12 years, 95% CI: −0.03, −0.04)^14^ to 0.10 (a 10% decrease in prevalence after the age of 12 years, 95% CI: 0.06, 0.15) (Figure 3).^20^ The overall mean weighted percentage difference in prevalence was not significantly different from 0 (0.01, a 1% decrease, 95% CI: 0.02, −0.050). We found similar results when we explored multiple age cutoffs in sensitivity analyses (Table S5), and Eggers test showed no significant publication bias (*P* = .314). Because of the small number of included studies, funnel plots were not created.

### 3.4 Heterogeneity

There was significant heterogeneity in the primary analysis (I^2^ 96.3%). Multiple sensitivity analyses were performed to explore the impact of study design features on the degree of heterogeneity and the outcome. First, we considered differences in the age structure of the cohorts: Ballardini et al's study only followed individuals through the age of 12 years, while all the other studies followed individuals through ages of 18‐26 years. Excluding this single study only reduced the I^2^ to 93.4%. Secondly, we considered the possibility of chronological trends: Williams et al's study included individuals who were born in 1958 (2‐3 decades prior to the other cohorts); excluding this study reduced the I^2^ to 81.9%. Next, we examined whether the disease definition impacted the heterogeneity: Finnobogadottir et al was the only study to require eczematous symptoms to have begun in early childhood, which would have excluded individuals with older‐onset disease from their AD classification and could have made the risk difference seem especially large. Excluding this study from the meta‐analysis did not change the I^2^; it remained at 96.3%. In all of these aforementioned analyses, our overall conclusion that there was no significant change in the prevalence of AD after the age of 12 years remained robust. Finally, we tested whether the origin of the study (UK vs non‐UK), and the number of times AD was measured (3‐4, 4‐5, or 5‐6) affected our estimates. In both cases, there were only mild reductions in heterogeneity (see Table S5 and Figures S1 and S2).

### 3.5 Risk of bias

The included studies had variable risk of bias with Newcastle‐Ottawa scores ranging from 3 to 7 of a total maximum score of 8. One of the criteria, demonstration that the outcome of interest (ie, AD diagnosis) was not present at the start of the study, was not relevant given that all of the included studies were birth cohorts. In terms of selection, most (7/9) cohorts were representative of the general population. The Gough et al and Burr et al studies included only healthy newborn children with family histories of atopic disease and therefore may overestimate AD prevalence. Moreover, the Burr cohort was followed after a randomized controlled trial of milk protein over the first 4 months of life, which may introduce a bias as families willing to participate in a RCT may be different from those who decline. The ascertainment of AD was sometimes self‐reported, but always based on a standardized questionnaire or examination applied to all participants (Table S6). In Burr et al's study, the definition of AD used varied over time and could have introduced bias: They measured the point prevalence of AD through age of 7 years, then annual period prevalence from ages 15 and 23 years, which could make AD rates appear higher during adulthood. A sensitivity analysis excluding this study did not change the results (Table S5). All studies had good comparability due to equivalent measurements between AD and non‐AD controls, and all studies had an adequate length of follow‐up. Loss to follow‐up was >30% for 3 of 7 studies; excluding these studies did not affect our results (Table S5 and Figure S3).

### 3.6 Analyses of repeated measures among individuals

Four studies examined whether individuals reporting AD symptoms at each age had a history of prior disease. All found that there was substantial turnover with many new and remitting cases throughout the study period. However, studies were inconsistent in providing study definitions of disease “remission” and “persistence” (Table S3). In 1 study, persistence was defined as AD symptoms at 2 or more observation points at any time within the study^18^; another required subjects to have AD symptoms over the course of all study assessments, and across consecutive observation points.^23^ This inconsistency in the definition of AD persistence and reporting makes it difficult to compare results across studies.

Multiple studies reported the proportion of patients with new‐onset disease at each age of measurement. Incidence rates were highest in early childhood, but remained above zero throughout the teens and early 20s. Williams et al found that of the 870 cases with examined or reported AD onset by the age of 16 years, 66% had onset by the age of 7 years. Nissen et al, found the highest proportions of new‐onset AD onset before the age of 1.5, and Ballardini et al reported that over all time periods, the total proportion of new (vs repeated cases) was 53%.

Multiple studies found that individuals with early‐onset AD were more likely to have symptoms at older ages. In Williams et al, patients with early‐onset AD by the age of 1 year were more likely to have AD at the age of 23 years (*P* < .001). Similar findings were presented by Burr et al, whereby AD onset by the age of 1 year was associated with AD at the age of 7 and 15 years, and for those with AD at 7 or 15 years, there was an association with AD at the age of 23 years. In Ziyab et al, of the patients that developed AD by the age of 2 years, 16.9% of patients had AD present at all time points up to the age of 18 years; by contrast, in children who developed AD by the age of 4 years, the proportion with AD at all time points was only 10.9%.

## 4 DISCUSSION

In this systematic review and meta‐analysis of 7 birth cohort studies including over 13 000 individuals, we found no significant difference in AD prevalence before and after childhood. Our finding fills a gap in the literature about AD in adolescence and early adulthood. It also highlights the importance of longitudinal analyses to understand the natural history of an episodic condition. In the studies included in our review that report on the consistency of individual responses over time, the presence of AD symptoms was variable. Individuals reported intermittent periods without symptoms followed by periods with symptoms again. Because studies did not consistently present repeated measures of disease activity or severity among individuals, we are unable to conclude whether AD tends to get better or worse over time. Nonetheless, the available data suggest that the reason for steady prevalence estimates across ages is due to a combination of active disease in both childhood and early adulthood among some individuals, remission or clearance of disease among others, and later‐onset disease among others.

### 4.1 Comparison with other studies

A recent review focused on “AD persistence” concluded that “80% did not persist by 8 years.”^24^ It measured the change in the average proportion of a population reporting AD symptoms upon second measurement at any age. Upon first glance, these findings may seem contrary to ours. However, the authors caveat that “it is possible that some of the patients reported to have AD remittance had unobserved recurrences later in life.” Thus, although their results are not directly comparable to ours because they included studies with variable populations, definitions of AD, and timing of follow‐up, their findings also highlight the episodic nature of the condition.

Our results concur with previous work showing high rates of AD persistence in a longitudinal cohort of children and young adults treated with pimecrolimus, which may be biased toward more persistent disease.^25^ This study did not meet inclusion criteria for this review because enrollment was clinic‐based and it included only patients with AD which did not allow for estimates of disease prevalence. The population‐based ISAAC studies calculated AD prevalence at 2 age points in sites around the world, but were cross‐sectional in nature and did not follow the same individuals over time. There was variation in prevalence by site and by age, but as with our findings, the average prevalence at both time points was similar (7.9% at the age of 6‐7 and 7.3% at the age of 13‐14).^26^

It is important to highlight that diagnostic definitions may impact prevalence estimates, possibly biasing toward smaller numbers at older ages. Notably, the 1 study that required symptom onset occur in “early childhood” for the definition of AD found the sharpest decline in prevalence,^20^ possibly because new‐onset cases at later ages were not included. Other, more commonly used definitions of AD, such as the classic Hanifin and Rajka criteria and the widely used UK Working Party Criteria, assign extra points if the AD diagnosis is made in early childhood.^11^, ^27^ Subsequently, studies using these criteria may report smaller numbers of individuals with later‐onset disease, which should be taken into account when considering estimates of adult AD.

### 4.2 Strengths and limitations

Major strengths of our review and meta‐analysis include the comprehensive search, careful selection and critical appraisal of studies, and inclusion of large representative cohorts followed from birth over 2‐3 decades. Of note, we did not specify the initial age of follow‐up in our review; it was coincidental that all of the studies that met inclusion criteria were designed as birth cohort studies.

Limitations also warrant discussion. First, there is variability in the terminology used to describe AD: “Atopic dermatitis,” “atopic eczema,” and “eczema” are all used to refer to the condition.^28^ In some settings, “eczema” is considered a less specific term that may include other types of dermatitis. Therefore, we carefully reviewed the diagnostic criteria used in each study (Table S3) and assessed the impact on our results as described above. Second, we found a high level of statistical heterogeneity between studies. This might be due in part to the use of a risk difference as our outcome measure, as risk differences are often less homogeneous.^16^, ^29^ We used a model with random effects to help account for the statistical heterogeneity, and found no appreciable differences in the results after multiple analyses to address differences in the methods for and timing of AD ascertainment between studies (Table S5). Nonetheless, our results should be interpreted with caution and replicated as additional follow‐up data from longitudinal cohorts become available. Third, most studies lacked important information on secondary outcomes including treatment use, disease severity, and other atopic conditions. These data may impact the clinical course of AD and are important to report. Fourth, as is common for studies that follow large cohorts of patients over decades, attrition was substantial, ranging from 10 to 53%. Most of the studies (7 of 9) were not designed to specifically study AD, and none were organized through clinic visits so dropout was likely random with respect to AD and would therefore be unlikely to bias prevalence estimates. However, other factors differentially associated with attrition such as race/ethnicity and socioeconomic status may also be associated with AD resulting in selection bias.^30^ These data should be reported in future longitudinal studies. Finally, all of the studies identified were from Northern European countries, and patients were only followed into the third decade of life. Additional work is needed to understand the clinical course throughout adulthood and in more diverse settings.

## 5 CONCLUSIONS AND IMPLICATIONS FOR PRACTICE AND RESEARCH

Our finding of similar AD prevalence before and after childhood supports the emerging paradigm of AD as a lifelong genetic predisposition to episodic skin lesions.^3^ Studies examining predictors of individual disease course are a high priority for additional research and can help to elucidate the relationship between genetic susceptibility and environmental influences on the epidemiological trends described herein.

## CONFLICT OF INTEREST

None reported.

## AUTHOR CONTRIBUTIONS

All authors were involved in the study design. Dr. Abuabara had full access to all of the data in the study and takes responsibility for the integrity of the data and the accuracy of the data analysis. Drs. Abuabara, Langan, and Allen involved in study concept and design. Yu and Okhovat involved in acquisition, analysis, and interpretation of data. Yu and Abuabara drafted the manuscript. All authors critically revised the manuscript for important intellectual content. Abuabara and Allen WERE involved in the statistical analysis. Abuabara obtained funding. None of the authors receive administrative, technical, or material support. Abuabara supervised the study.
