# Supplementary material for: The prevalence of atopic dermatitis beyond childhood: A systematic review and meta‐analysis of longitudinal studies
Source: Allergy. 2017 Nov 24;73(3):696–704. doi: 10.1111/all.13320 (PMC5830308; doi:10.1111/all.13320)

**Table S1.** Pubmed Search

| **Search** | **Search Terms** | **Results** |
| --- | --- | --- |
| infancy/ childhood | ("Dermatitis, Atopic"[Mesh] OR "Eczema"[Mesh] OR atopic dermatitis[tw] OR eczema[tw]) AND ("Infant"[Mesh] OR "Child"[Mesh] OR birth[tw] OR infants[tw] OR infancy[tw] OR neonates[tw] OR neonatal[tw] OR newborn[tw] OR newborns[tw] OR new born*[tw] OR preschool[tw] OR child[tw] OR children[tw] OR childhood[tw] OR youth[tw] OR youths[tw]) | 12,341 |
| adolescence/ adulthood | ("Dermatitis, Atopic"[Mesh] OR "Eczema"[Mesh] OR atopic dermatitis[tw] OR eczema[tw]) AND ("Adolescent"[Mesh] OR "Adult"[Mesh] OR adolescent[tiab] OR adolescents[tiab] OR adolescence[tiab] OR teenagers[tiab] OR teen[tiab] OR teens[tiab] OR teenage[tiab] OR adult[tw] OR adults[tw] OR adulthood[tw] OR 18 years[tw]) | 14,044 |
| longitudinal/ follow up/ natural history | ("Dermatitis, Atopic"[Mesh] OR "Eczema"[Mesh] OR atopic dermatitis[tw] OR eczema[tw]) AND ("Follow-Up Studies"[Mesh] OR "Longitudinal Studies"[Mesh] OR "Age Factors"[Mesh] OR "Recurrence"[Mesh] OR year follow up[tw] OR natural history[tw] OR longitudinal[tw] OR follow up stud*[tw] OR followup stud*[tw] OR long term follow up[tw] OR to adulthood[tw] OR persist[tw] OR persisted[tw] OR persistence[tw] OR over time[tw] OR recurrent[tw] OR recurrence[tw] OR recurrences[tw] OR recurring[tw] OR recur[tw] OR remission[tw]) | 4,098 |
| epidemiology/ prevalence/ population/ birth cohort | ("Dermatitis, Atopic"[Mesh] OR "Eczema"[Mesh] OR atopic dermatitis[tw] OR eczema[tw]) AND ("Prevalence"[Mesh] OR "epidemiology" [Subheading:NoExp] OR epidemiology[tw] OR epidemiologic[tw] OR epidemiological[tw] OR epidemiology[tw] OR prevalence[tw] OR population based[tw] OR birth cohort*[tw]) | 5,967 |

**Table S2**. Risk of bias assessment: Newcastle-Ottawa Scale

| **SELECTION** | |
| --- | --- |
| 1 | Representativeness of the exposed cohort (maximum of 1 point):   - 1 point if the study cohort is representative of other people from the same study community (i.e. general population) - 1 point if the study cohort is somewhat representative of other people from the same study community - 0 points if the study cohort was a selected subgroup of the population (i.e. only asthma patients, only males, etc.) - 0 points if there is no description of the derivation of the cohort |
| 2 | Selection of the non-exposed cohort (maximum of 1 point):   - 1 point if the non-eczema cohort is drawn from the same population as the eczema patients - 0 points if the non-eczema cohort is drawn from a different source - 0 points if there is no description of the derivation of the non-eczema cohort |
| 3 | Ascertainment of exposure (maximum of 1 point):   - 1 point if ascertainment of eczema outcome was from a secure record or structured interview - 0 points if ascertainment of previous eczema from written self reports - 0 points if there is no description on the ascertainment of eczema |
| 4 | Demonstration that outcome of interest (i.e. eczema diagnosis) was not present at the start of the study:   - 1 point if eczema not present at study start - 0 points if not described |
| **COMPARABILITY** | |
| 1 | Comparability of cohorts on the basis of design or analysis (maximum of 2 points):   - 1 point if the study controls (non-eczema) are comparable to eczema patients at each time point - 1 point if the study controls for additional factors when comparing eczema and non-eczema patients at each time point (i.e. patients lost to follow up) |
| **OUTCOME** | |
| 1 | Assessment of outcome (maximum of 1 point):   - 1 point if stated that independent blinded assessments or record linkage occurred to ascertain eczema outcomes (i.e. outcome assessors unaware of study hypothesis at time of assessment) - 0 points if outcome of eczema was self reported - 0 points if method of ascertainment of eczema outcomes was not described |
| 2 | Was follow-up long enough for outcomes to occur (maximum of 1 point):   - 1 point if length of follow-up was adequate (> 1 year) to ascertain outcome of eczema at each time point - 0 points if length of follow-up was not adequate to ascertain outcome of eczema |
| 3 | Adequacy of follow up of cohorts (maximum of 1 point):   - 1 point if there was adequate follow up with no patients lost to follow up - 1 point if there was adequate follow up with <30% patients lost to follow up and a description of those patients that were lost to follow up - 0 points if on average, <30% of patients were lost to follow up and there was no description of those that were lost - 0 points if on average, >30% of patients were lost to follow up - 0 points if there is no statement about how many patients were lost to follow up and why |

**Table S3**. Study definitions of eczema, remission, and persistence

| **Study** | **Study definition of eczema (study diagnostic criteria)** | **Study definition of remission** | **Study definition of persistence** |
| --- | --- | --- | --- |
| Ballardini et al., 2012^18^ | Dry skin, itchy rashes, with age-specific location for 2 weeks or more and/or doctor's diagnosis of eczema in the past 12 months. | Total remission: never having a specific allergy-related disease again that had been present at the previous follow-up. Remission and relapse: not having a specific allergy- related disease that had been present at the previous follow-up and that will be present at one or more future follow-ups. | The proportion of children who had disease at one, two, three, or more observation points among the children who had ever had the same disease. |
| Burr et al., 2013^19^ | Children were examined by a doctor up to age 7 years, rashes were classified as noneczematous rash, a history of eczema, inactive eczema, and active eczema. At age 15 and 23 years, subjects were sent a short questionnaire including questions about eczema (from the ISAAC study).^14^ | Not reported | Not reported |
| Finnbogadóttir et al., 2012^20^ | Recurrent or continuous erythema, scaling, and itching in typical places, the symptoms starting in early childhood. | Not reported | Not reported |
| Gough et al., 2012^21^ | An itchy rash that persisted for at least 6 months and was located in the antecubital or popliteal fossae, wrists, ankles, neck or face during the last 12 months (from the ISAAC study). | Not reported | Not reported |
| Nissen et al., 2013^22^ | Areas of scaly, erythematous, and itchy eczematous rash primarily of the face and scalp, behind the ears, and at the flexural folds, diagnosed by a doctor. Only eczema localized to at least two typical areas and chronically relapsing with duration of at least 3 months were recorded. | Defined as not having the disease that had been present at the previous study  period(s). | Indicates the proportion of participants who had a disease in one (I), two (II), three (III), four (IV) or five (V) consecutive study periods among participants who had ever had the same disease. |
| Williams et al., 1998^14^ | When the children were aged 7 years, parents were asked by health visitors using a “structured questionnaire” whether their child had had any eczematous rash during the first year of life or at any time after the first year. When the children were aged 11 or 16 years, parents were asked whether their child had had eczematous rashes in the past 12 months. Self- reported eczema in the last 12 months at age 23 years was also recorded. The presence of visible eczema was recorded by experienced school medical officers at the ages of 7, 11 and 16 years. | By means of a combination of visible eczema at one point and reported symptoms over the last year in order to capture both the doctors’ and the parents’ perspective in this chronic relapsing disease | Not reported |
| Ziyab et al., 2010^23^ | Defined as ‘ever [having] eczema’ plus an ‘itchy rash during the previous 12 months. Atopic eczema was defined as having both eczema and a positive skin prick test. | A change in the individual status from eczema to eczema free. | Persistent cases were those present over the course of all assessments (ages 1-or-2, 4, 10, and 18 years), or those from 4 years and onwards. |

**Table S4**. Prevalence data from included studies

| **Reference** | **3 mo** | **6 mo** | **1**  **yr** | **2 yrs** | **3 yrs** | **4 yrs** | **5 yrs** | **6 yrs** | **7 yrs** | **8 yrs** | **9 yrs** | **10 yrs** | **11 yrs** | **12 yrs** | **15 yrs** | **16 yrs** | **18 yrs** | **20 yrs** | **21 yrs** | **23 yrs** | **26 yrs** |
| --- | --- | --- | --- | --- | --- | --- | --- | --- | --- | --- | --- | --- | --- | --- | --- | --- | --- | --- | --- | --- | --- |
| Ballardini et al^18^ |  |  | $\frac{440}{2916}$ | $\frac{542}{2916}$ |  | $\frac{546}{2916}$ |  |  |  | $\frac{355}{2916}$ |  |  |  | $\frac{339}{2916}$ |  |  |  |  |  |  |  |
| Burr et al^19^ | $\frac{37}{391}$ | $\frac{44}{378}$ | $\frac{53}{378}$ |  |  |  |  |  | $\frac{26}{342}$ |  |  |  |  |  | $\frac{30}{283}$ |  |  |  |  | $\frac{33}{241}$ |  |
| Finnbogadóttir et al^20^ |  |  |  | $\frac{55}{179}$ |  | $\frac{34}{161}$ |  |  |  | $\frac{27}{134}$ |  |  |  |  |  | $\frac{16}{122}$ |  |  | $\frac{10}{120}$ |  |  |
| Gough et al^21^ |  |  |  |  | $\frac{19}{200}$ |  |  | $\frac{24}{200}$ |  |  | $\frac{34}{200}$ |  |  | $\frac{34}{200}$ | $\frac{31}{200}$ |  |  | $\frac{26}{200}$ |  |  |  |
| Nissen et al^22^ |  |  |  | $\frac{36}{276}$ |  |  | $\frac{23}{251}$ |  |  |  |  | $\frac{24}{222}$ |  |  | $\frac{21}{215}$ |  |  |  |  |  | $\frac{11}{193}$ |
| Williams et al^14^ |  |  |  |  |  |  |  |  | $\frac{571}{6877}$ |  |  |  |  |  |  | $\frac{860}{6877}$ |  |  |  | $\frac{1053}{6877}$ |  |
| Ziyab et al^23^ |  |  |  | $\frac{196}{1377}$ |  | $\frac{145}{1214}$ |  |  |  |  |  | $\frac{186}{1359}$ |  |  |  |  | $\frac{161}{1307}$ |  |  |  |  |

*Figure legend: mo: months; yr: year; yrs: years; when a range of years was given, numbers were rounded up*

**Table S5.** Results of sensitivity analyses

| ***By cutoff age*** | **Overall weighted risk difference** | **95% confidence interval** | | **I^2^** |
| --- | --- | --- | --- | --- |
| Under and over age 10 | 0.014 | -0.022 | 0.049 | 96.30% |
| Under and over age 8 | 0.006 | -0.031 | 0.044 | 97.10% |
| Under and over age 2* | 0.014 | -0.004 | 0.032 | 62.20% |
| Under and over age 12 (excluding those under age 2) | 0.002 | -0.032 | 0.036 | 94.90% |
| ***By Country*** |  |  |  |  |
| Other | 0.034 | -0.001 | 0.068 | 82.20% |
| UK | -0.017 | -0.055 | 0.022 | 93.40% |
| ***By number of assessments*** |  |  |  |  |
| 3-4 Assessments | -0.019 | -0.072 | 0.033 | 96.30% |
| 5 Assessments | 0.049 | 0.015 | 0.082 | 74.80% |
| 6 Assessments | -0.013 | -0.036 | 0.009 | 0.00% |
| ***Sensitivity Analysis - excluding one study at a time*** |  |  |  |  |
| No Ballardini | 0.007 | -0.028 | 0.043 | 93.40% |
| No Burr | 0.016 | -0.025 | 0.057 | 96.90% |
| No Finnbogadottir | -0.001 | -0.037 | 0.036 | 96.30% |
| No Gough | 0.017 | -0.023 | 0.057 | 96.90% |
| No Nissen | 0.010 | -0.030 | 0.049 | 96.70% |
| No Williams | 0.021 | -0.003 | 0.045 | 81.90% |
| No Ziyab | 0.013 | -0.030 | 0.056 | 96.80% |

Notes:* Gough and Williams studies excluded because they did not include children under age 2

**Table S6.** Newcastle-Ottawa Scale Risk of Bias Assessment

| **Author, year** | **Selection** | | | | **Comparability** | | **Outcome Assessment** | | | **Total Score** |
| --- | --- | --- | --- | --- | --- | --- | --- | --- | --- | --- |
|  | **Representa-tiveness of cohort** | **Selection of non-exposed** | **Ascertain-ment of exposure** | **Outcome not present at start** | **Controls for factor** | **Additi-onal factors** | **Assessm-ent of outcome** | **Length of follow-up** | **Adequacy of follow-up** |  |
| Ballardini et al.^18^ | √ | √ | †† | †† | √ | †† | †† | √ | √ | 5 |
| Burr et al..^19^ | †† | †† | √ | †† | √ | †† | †† | √ | †† | 3 |
| Finnbogadóttir et al.^20^ | √ | √ | √ | †† | √ | †† | √ | √ | †† | 6 |
| Gough et al.^21^ | †† | †† | √ | †† | √ | †† | †† | √ | √ | 4 |
| Nissen et al.^22^ | √ | √ | √ | †† | √ | †† | √ | √ | √ | 7 |
| Williams et al.^14^ | √ | √ | √ | †† | √ | †† | †† | √ | †† | 5 |
| Ziyab et al.^23^ | √ | √ | †† | †† | √ | †† | †† | √ | √ | 5 |

√ No risk of bias (1 point), †† Risk of bias or unclear risk

**Figure S1.** Results of sensitivity analysis by country

**Figure S2.** Results of sensitivity analyses by the number of assessments in each study

**Figure S3**. Results of sensitivity analysis including only studies with <30% follow-up


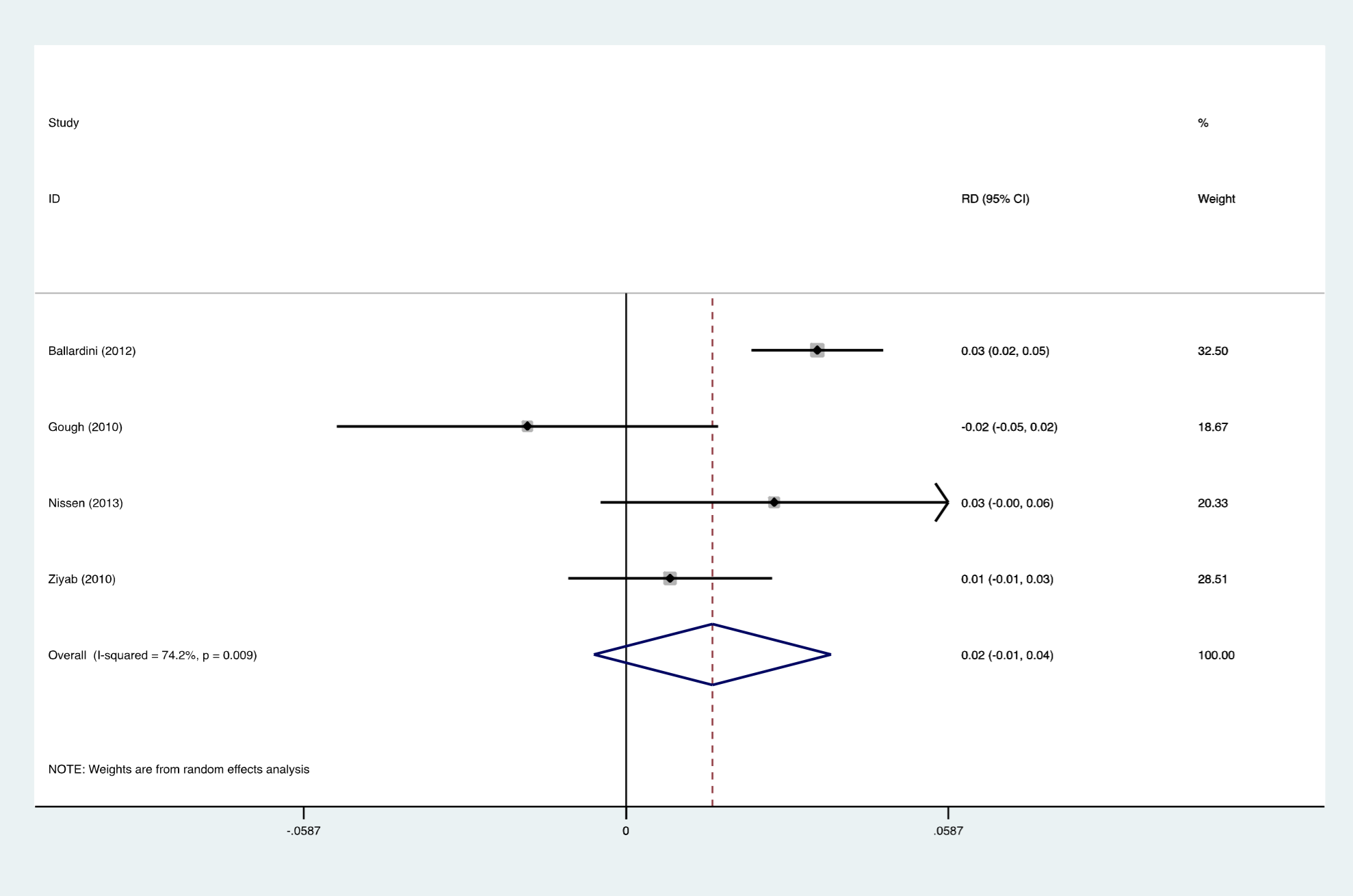

Supplement: Supplementary file 1 [file ALL-73-696-s001.docx]
